# Mutations in adaptively evolved *Escherichia coli* LGE2 facilitated the cost-effective upgrading of undetoxified bio-oil to bioethanol fuel

**DOI:** 10.1186/s40643-021-00459-2

**Published:** 2021-10-22

**Authors:** Dongdong Chang, Cong Wang, Fabrice Ndayisenga, Zhisheng Yu

**Affiliations:** 1 College of Resources and Environment, University of Chinese Academy of Sciences, Beijing, 100049 People’s Republic of China; 2 RCEES-IMCAS-UCAS Joint-Lab of Microbial Technology for Environmental Science, Beijing, 100085 People’s Republic of China

**Keywords:** Levoglucosan, Bioethanol, *Escherichia coli*, Inhibitor tolerance, Evolution, Whole-genome sequencing

## Abstract

Levoglucosan is a promising sugar present in the lignocellulose pyrolysis bio-oil, which is a renewable and environment-friendly source for various value-added productions. Although many microbial catalysts have been engineered to produce biofuels and chemicals from levoglucosan, the demerits that these biocatalysts can only utilize pure levoglucosan while inhibited by the inhibitors co-existing with levoglucosan in the bio-oil have greatly limited the industrial-scale application of these biocatalysts in lignocellulose biorefinery. In this study, the previously engineered *Escherichia coli* LGE2 was evolved for enhanced inhibitor tolerance using long-term adaptive evolution under the stress of multiple inhibitors and finally, a stable mutant *E. coli*-H was obtained after ~ 374 generations’ evolution. In the bio-oil media with an extremely acidic pH of 3.1, *E. coli*-H with high inhibitor tolerance exhibited remarkable levoglucosan consumption and ethanol production abilities comparable to the control, while the growth of the non-evolved strain was completely blocked even when the pH was adjusted to 7.0. Finally, 8.4 g/L ethanol was achieved by *E. coli*-H in the undetoxified bio-oil media with ~ 2.0% (w/v) levoglucosan, reaching 82% of the theoretical yield. Whole-genome re-sequencing to monitor the acquisition of mutations identified 4 new mutations within the globally regulatory genes *rssB*, *yqhA*, and *basR,* and the − 10 box of the putative promoter of *yqhD*-*dgkA* operon. Especially, *yqhA* was the first time to be revealed as a gene responsible for inhibitor tolerance. The mutations were all responsible for improved fitness, while *basR* mutation greatly contributed to the fitness improvement of *E. coli*-H. This study, for the first time, generated an inhibitor-tolerant levoglucosan-utilizing strain that could produce cost-effective bioethanol from the toxic bio-oil without detoxification process, and provided important experimental evidence and valuable genetic/proteinic information for the development of other robust microbial platforms involved in lignocellulose biorefining processes.

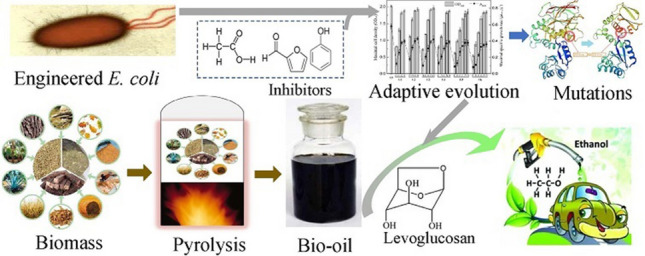

## Introduction

As concerns on the environmental and energy issues are increasing due to the massive usage of fossil fuels, the development of bio-based systems for the production of biofuels and chemicals from renewable resources that serve as a replacement for unsustainable fossil fuels has become more and more attractive (Mat Aron et al. 2020). Lignocellulosic biomass is the largest source of renewable and sustainable carbon-based energy on our planet (Itabaiana Junior et al. 2020); however, the utilization efficiency of lignocellulosic biomass is far from our expectations because lignocellulose is difficult to be bioconverted to high value-added productions before depolymerization (Zhao et al. 2019).

Pyrolysis process that does not require any solvent or enzyme to depolymerize lignocellulose can easily and cheaply decompose lignocellulose to composition-complex bio-oil, which mainly contains levoglucosan (1,6-anhydro β-d-glucopyranose), a promising six-carbon sugar substrate (Anex et al. 2010; Itabaiana Junior et al. 2020). Levoglucosan can further be bioconverted to various biofuels and chemicals by different engineered microorganisms (Kim et al. 2015; Layton et al. 2011; Linger et al. 2016; Xiong et al. 2016) introduced with a heterologous gene *lgk* from *Lipomyces starkeyi* (Dai et al. 2009). However, these investigated studies all focused on the bioconversion of pure levoglucosan that is purified from the bio-oil; and this purification step greatly increases the overall cost and process complexity which make the approach not economically feasible for its industrial implementation. Furthermore, direct microbial fermentation of levoglucosan-containing bio-oil is also impractical owing to the existence of many bio-toxic molecules like organic acids, furans, and phenols that can prevent microbial growth and fermentation (Islam et al. 2015). Although physical and/or chemical detoxification processes can remove the bio-toxic chemicals co-existing with levoglucosan and make the treated bio-oil fermentable (Chi et al. 2013; Rover et al. 2014), this detoxification step also becomes a major challenge toward its commercialization since it makes the whole conversion process more costly and complex.

The most ideal and economically feasible way to make the bio-oil fermentable is to develop robust strains that can innately tolerate or utilize most of the bio-toxic substances, avoiding the extra use of purification or detoxification process. In this regard, genetic engineering strategies can be pursued to develop robust inhibitor-tolerant strains (Choi et al. 2015; Sasano et al. 2012; Wang et al. 2013). However, strong cellular tolerance toward inhibitors often results from multiple genetic changes rather than one or two changes (Thorwall et al. 2020). Therefore, adaptive evolution, as the result of polygenic responses that could confer plenty of beneficial properties like improved cell growth, cell viability, and product productivity on the strain under stress conditions over the time of evolution (Arense et al. 2010; Thorwall et al. 2020; Wang et al. 2018; Yan et al. 2021), might be a more suitable strategy to improve the cellular tolerance toward the complex lignocellulose-derived inhibitors.

In addition, re-sequencing the whole genome of the evolved and non-evolved individuals of the same species to identify the single-nucleotide polymorphisms (SNPs) is essential to determine the genomic sequence variations after the directed evolution (Henry et al. 2011; Otero et al. 2010). Especially, the comparative SNPs analysis between the evolved strain and its parent could reveal what genetic changes have made the strain a better one with desired properties (Herring et al. 2006; Maeda et al. 2020). Therefore, SNPs analysis via genomic re-sequencing is a powerful tool for us to understand the relationship between the phenotype and genotype, and the corresponding mutation database obtained by the analysis could further guide us to genetically modify the targeted biological pathway of commonly used microbial platforms for efficient microbial cell factories’ design.

To the best of our knowledge, there has been no investigation on improving the phenotypic robustness of the levoglucosan-utilizing strain against lignocellulosic inhibitors till now, although a few attempts have been pursued to develop non-levoglucosan-utilizing strains tolerant to lignocellulosic inhibitors by targeted and evolutionary approaches (Choi et al. 2015; Jin et al. 2019; Kurosawa et al. 2015; Liu et al. 2020; Sasano et al. 2012; Shah et al. 2020; Wang et al. 2013). Therefore, this is the first-ever study that developed a robust *E. coli* strain for the economically efficient bioethanol production from undetoxified lignocellulosic bio-oil by using an adaptive evolution strategy. The evolved strain could tolerate inhibitors commonly found in lignocellulosic bio-oil, exhibiting much-improved inhibitor tolerance. Furthermore, the current study also revealed the genetic traits of the evolved strains that could help in understanding the adaption nature of the obtained phenotypes against the harsh conditions of bio-oil, paving ways toward the development of more robust strains that could directly produce bioethanol from the biomass-derived levoglucosan substrate. This is the first study toward resolving the inhibitor issue for the in situ upgrading of bio-oil by inhibitor-tolerant biocatalyst rather than by detoxification method.

## Experimental

### Bacterial strains and growth media

Strains used in this study are listed in Table 1. The levoglucosan-utilizing and ethanol-producing strain *E. coli* LGE2 we previously constructed (Chang et al. 2021), were used as the starting strain. LB media (10 g/L tryptone, 5 g/L yeast extract, and 5 g/L NaCl) were used for the laboratory adaptive evolution. Bio-oil-based M9 minimal media (7.10 g/L Na_2_HPO_4_, 3.00 g/L KH_2_PO_4_, 0.50 g/L NaCl, 1.00 g/L NH_4_Cl, 0.49 g/L MgSO_4_, 14.70 mg/L CaCl_2_, and ~ 11% pyrolytic bio-oil) were used for ethanol fermentation from bio-oil. Ampicillin, chloramphenicol, and isopropyl β-d-1-thiogalactopyranoside (IPTG) with a final concentration of respective 100 μg/mL, 34 μg/mL, and 0.06 mM were added into the used aforementioned media.

**Table 1:** Strains used in this work

Strains	Description	Source
*E. coli* LGE2	F^–^ *omp*T *gal dcm lon hsd*SB (rB^−^ mB^−^) λ(DE3) *lgk pdc adh* Amp^r^ Cm^r^	Laboratory collection
*E. coli*-L	302 generations evolved from *E. coli* LGE2	This study
*E. coli*-H	72 generations evolved from *E. coli*-L	This study

Underlined regions represent heterologous genes

### Laboratory evolution

To initiate laboratory evolution, the *E. coli* LGE2 strain was cultivated microaerobically in a 250-ml unbaffled shake flask containing 100 ml LB medium without the inhibitors in an incubator with the orbital shaker set at 150 rpm overnight, the controlled temperature was set at 37 °C and then reserved as the seed culture. Afterward, 1% (v/v) of the seed culture was inoculated into a fresh LB medium added with the inhibitors; the initial OD_600_ value was detected as ~ 0.06. The inhibitors were a mixture of acetic acid, furfural, and phenol, which represent the representative inhibitors derived from the pretreatment of biomass (Chi et al. 2013; Islam et al. 2015; Li et al. 2019).

According to the minimal inhibitory concentration (MIC) and half-inhibitory concentration (IC_50_) values of these inhibitors identified for *E. coli* LGE2 in the preliminary experiments, 0.7 g/L acetic acid, 0.8 g/L furfural, and 0.6 g/L phenol were together added to the LB medium for the first-round evolution, resulting in a pH value of 4.5. Then for each round of evolution, the last-round evolved strain was transferred to a fresh LB medium, with the inhibitor concentration increasing by 20% of their initial added concentration, that is, an increment of 0.14 g/L acetic acid, 0.16 furfural g/L, and 0.12 g/L phenol. This increment was defined as one concentration increase value (CIV). The media pH was gradually decreasing from 4.5 to 3.6 with the increasing concentration of inhibitors. Every 48 h of cultivation, cell growth was monitored by measuring the OD_600_ of each culture, and an OD_600_  > 0.10 indicated cell growth. Triplicate cultures were evolved concurrently under identical conditions. The serial passage process for a certain concentration combination of the inhibitors was carried out with four to six batches until a stable growth rate was observed. The stable cultures were further streaked onto plates added with different concentrations of inhibitors corresponding to the concentration used in the relevant batch, to isolate pure colonies with the largest width and length from each evolution experiment. The selected colony from the plate was considered as a phenotypic and genotypic representative within the population. Finally, the adaptive evolution experiments were performed with a total of 95 days of serial transfers in liquid and solid media and the total number of generations was ~ 374.

### Preparation of genomic DNA

The cells were collected by centrifugation at 10,000 rpm for 5 min and the cell pellets were stored at − 80 °C before genomic DNA purification. Genomic DNA was isolated and purified using a PureLink microbiome DNA purification kit (Invitrogen) following the manufacturer’s instructions. The quantity and purity of the genomic DNA were determined by measuring the absorbance at 260 nm and calculating the ratio of absorbance at 260 and 280 nm (A260/280) using a NanoDrop ND-2000 spectrophotometer, respectively. The A260/280 values of all the samples were confirmed to be greater than 1.7. The purified genomic DNAs were stored at − 20 °C before use.

### Genome sequencing using Illumina HiSeq system

Whole-genome re-sequencing was performed using a combination of PacBio RS II and Illumina Hiseq sequencing platforms (Majorbio Bio-Pharm Technology Co., Ltd, Shanghai, CN). For Illumina sequencing, at least 1 mg genomic DNA was utilized for assembly of the sequencing library. DNA fragments were incised into ~ 400 bp by a Covaris M220 Focused Acoustic Shearer. Firstly, 50 prime ends were end-repaired and phosphorylated, then 30 ends were A-tailed and ligated to sequencing adapters, and finally, the adapters-ligated products were enriched. The organized libraries were used for paired-end Illumina sequencing (2 × 150 bp) on an Illumina HiSeq X Ten. For PacBio sequencing, an aliquot of 15 mg DNA was spun in a Covaris G-tube at 6000 r/min for 60 s. DNA fragments were then purified, end-repaired, and ligated with SMRTbell sequencing adapters. The resulting sequencing library was sequenced on one SMRT cell using standard methods. The complete genome was assembled using both the PacBio and Illumina reads; the PacBio reads were assembled into a contig using HGAP and Canu, and the Illumina reads were used for the error correction of the PacBio assembly results using Pilon. In this study, *E. coli*-L and *E. coli*-H obtained by adaptive evolution were subjected to sequencing. Triplicate cultures of each strain were mixed and sequenced, which resulted in 5.53 and 5.79 Gbp of clean data to reach ~ 198- and 235-fold depth of coverage.

### Genome sequence analysis

Genome DNA sequence comparison was performed using BLASTN (http://blast.ncbi.nlm.nih.gov/Blast.cgi) in the GenBank database. Snippy 4.6.0 was used to detect single-nucleotide polymorphism (SNP), multiple nucleotide polymorphisms (MNP), insertion (INS), deletion (DEL), and a combination thereof (MIXED). SnpEff was used for the annotation of the mutation sites. All the intragenic mutations that could cause amino acid substitutions and intergenic mutations were confirmed by PCR amplification and followed by Sanger sequencing. Sequence data of strain *E. coli*-L and *E. coli*-H were submitted at the NCBI Sequence Read Archive (http://www.ncbi.nlm.nih.gov/sra) with BioProject ID PRJNA753234.

### Ethanol fermentation of the undetoxified bio-oil

Pyrolytic bio-oil derived from waste cotton was prepared as previously described (Chang et al. 2015). In addition to levoglucosan, many other compounds like formic acid, acetic acid, propionic acid, acetol, methyl acetate, furfural, 5-hydroxymethylfurfural (5-HMF), and maltol are also present in the bio-oil (Chang et al. 2015) and most of them are considered as potential inhibitors. Because of the unavailability of most inhibitor standards, we only quantitatively measured the concentrations of several representative inhibitors; for example, formic acid, acetic acid, furfural, and 5-HMF were detected as 2.8, 11.4, 9.3, and 15.2 g/L, respectively. The pH of pyrolytic bio-oil was ~ 2.3. The evolved strains *E. coli*-L and *E. coli*-H were used as the production strain. According to the levoglucosan concentration in the bio-oil, 11% (v/v) bio-oil as the sole carbon source was added into the M9 minimal medium, resulting in a final levoglucosan concentration of ~ 20 g/L in the fermentation medium with a pH of 3.1. Strains were pre-cultured overnight in 100 mL LB medium (pH of 7.0) in a 250-mL flask at 37 °C and 150 rpm, and then 1 mL of this pre-cultured cell suspension was inoculated into 100 mL fresh bio-oil containing M9 minimal media for fermentation at 30 °C and 150 rpm microaerobically. If needed, the medium pH was adjusted by Ca(OH)_2_. For each sampling time point during the 36-h cultivation, a 5-mL sample was taken for measuring levoglucosan, ethanol, formic acid, acetic acid, furfural, 5-HMF, and phenol concentration. All the fermentation tests were performed in triplicates.

### Analysis methods

The culture samples were centrifuged at 10,000 rpm for 10 min, and the cell pellets were washed and suspended in PBS solution (pH 7.0) for the detection of cell density. Cell density was estimated by measuring the optical density of suspension at 600 nm with a spectrophotometer (DU 800 Beckman Coulter). The supernatant of the culture sample was filtered through a 0.2-μm syringe filter for subsequent HPLC analysis. The levoglucosan, ethanol, formic acid, and acetic acid were analyzed by high-performance liquid chromatography (HPLC, LC-20AT, Shimadzu Corp.) equipped with an ion-exchange column (Transgenomic ICSep ICE-ION-300, 7.8 mm × 300 mm) and a refractive index detector (RID10A, Shimadzu Corp.). A mobile phase of 0.00085 N H_2_SO_4_ solution at a 0.4 mL/min rate was used and the column was operated at 58 °C. Furfural and 5-HMF were analyzed by HPLC equipped with a C18 column (250 mm × 4.6 mm, 5 μm particle size, Thermo Scientific Corp.) and a UV absorbance detector (SPD-20A). The used mobile phase was 80% methanol aqueous solution and the operating temperature was room temperature. Three-dimensional modeling of the mutated proteins was performed by the SWISS-MODEL tool (http://swissmodel.expasy.org/) and the downloaded PDB files were modified by Chimera 1.15 software.

### Statistical analysis

The function of Student’s *t*-test (two-tailed distribution and two-sample unequal variance) in OriginPro 9.0 software was used to analyze the statistical difference between two arrays of data obtained in the above experiments. If the returned *p*-value is less than 0.05, the difference was considered significant.

## Results and discussion

### Adaptive evolution generated highly inhibitor-tolerant strain

An evolutionary engineering approach based on long-term adaptation in lignocellulose-derived inhibitors to select strains for enhanced inhibitor tolerance was envisaged in the current study. Our previously engineered strain *E. coli* LGE2 (Chang et al. 2021) was chosen for the evolution experiment to screen for robust strains with genetic changes that are linked to the cellular tolerance to the representative lignocellulosic inhibitors-acetic acid, furfural, and phenol. Growth of *E. coli* LGE2 was first evaluated in LB media with increasing concentrations of inhibitors. About 2.1 g/L acetic acid, 2.2 g/L furfural, and 1.8 g/L phenol were determined as the MIC of these inhibitors for *E. coli* LGE2, as no cell growth was observed after 48-h of cultivation in the presence of the inhibitors with these concentrations. Cultures in the presence of the combination of 0.7 g/L acetic acid, 0.8 g/L furfural, and 0.6 g/L phenol (~ 30% of their respective MIC) exhibited a long lag phase and low growth rate compared to those in inhibitor-absent LB media, while an increment of one CIV with 0.14 g/L acetic acid, 0.16 furfural g/L, and 0.12 g/L phenol completely abolished the cell growth. This implied that 30% of MIC of the inhibitors, which would impose significant pressure on the cell growth without completely blocking it, were suitable to be chosen as the starting inhibitory concentration of the inhibitor cocktail for strain evolution.

**Fig. 1:**
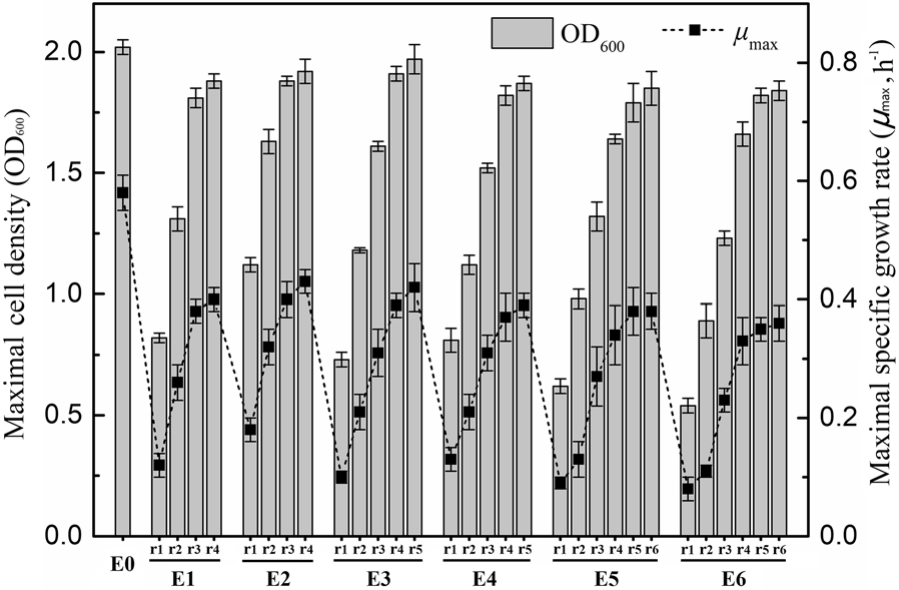
Comparison of the maximal cell density and specific growth rate of strains evolved under the stress of different concentrations of inhibitors. E0 represents the starting strain *E. coli* LGE2 growing in LB media without inhibitors. r1–r4 of E1 represent the strains that were evolved by four-round transfers in the same concentration of inhibitor cocktail composed of 0.7 g/L acetic acid, 0.8 g/L furfural, and 0.6 g/L phenol. For E2, the inhibitor concentration was increased by 0.14 g/L acetic acid, 0.16 furfural g/L, and 0.12 g/L phenol compared to E1, and this is also the increment of E3 vs E2, E4 vs E3, E5 vs E4, and E6 vs E5. r6 represents the strains that were successively transferred by six rounds. Each strain used in the new transfer was the strain obtained by the last-round evolution. These strains were all cultured microaerobically within a maximum of 96 h at 37 °C until the cells stopped growing

For each concentration of the inhibitor cocktail, at least four rounds of batch cultivation were performed to obtain the evolved strain with close final cell density to the control (Fig. 1). The first-round evolution with 0.7 g/L acetic acid, 0.8 g/L furfural, and 0.6 g/L phenol resulted in a cell density of ~ 0.82 within 48-h of incubation, while in the second-, third-, and fourth-round evolution the maximal OD_600_ of cells reached ~ 1.31, 1.81 and 1.94 within 48-h of incubation, respectively (E1 in Fig. 1). Especially, in the fourth-round evolution, cells grew significantly faster than the first-round cells (*p* < 0.01), with the maximal cell density observed at the 25-h time point. The improved cell growth rate implied the strain was evolved in the inhibitor cocktail. Subsequently, under the increasing inhibitory stress exposed by gradually improved inhibitor concentration, the strain was successfully evolved with enhanced inhibitor tolerance (Fig. 1). It is noteworthy that, when it was exposed to the inhibitor concentrations directly increased by two CIVs, the previous-round evolved strain showed no growth. This phenomenon was exhibited by all the evolved strains, suggesting that a moderate increase in inhibitor concentration was required for the gradual evolution and a sudden increase would impair the cell growth. Finally, the second-to-last evolved strain that was designed as *E. coli*-L could tolerate the inhibitor cocktail composed of 1.26 g/L acetic acid, 1.44 g/L furfural, and 1.08 g/L phenol and grow to a maximal cell density (OD_600_) of 1.85 within 24 h. The last evolved strain designed as *E. coli*-H could tolerate and grow in the presence of 1.4 g/L acetic acid, 1.6 g/L furfural, and 1.2 g/L phenol within 24 h of incubation, with the maximal cell density (OD_600_) reaching 1.82.

There are various bio-toxic inhibitors present in the lignocellulose-derived bio-oil, while the acetic acid, furfural, and phenol are three representative inhibitors that not only have toxicity themselves, but also have synergistic toxicity with other inhibitors (Chi et al. 2013; Islam et al. 2015; Li et al. 2019). A review has summarized that many publications have generated mutated *E. coli* with high acetate tolerance by adapting *E. coli* cells to different concentrations of acetate (Mills et al. 2009), but the used initial medium pH was often adjusted to a neutral or less acidic value. However, in this study, we did not adjust the initial pH before adaptation, because the low pH is consistent with the extremely acidic environment of the real bio-oil. The toxicity of acetic acid is strongly related to the concentration of the undissociated form, which is immensely affected by the external pH. It has been reported that the ethanol production titer and cell growth of ethanologenic *E. coli* in LB media decreased with the decreasing initial pH (Mills et al. 2009), and exposure of *E. coli* to moderate pH (5.0) before transferring to extremely low pH (3.0) can improve the cellular tolerance more than 50-fold (Goodson and Rowbury 2010). This implies that adaptive evolution is a good strategy for the improvement of cellular acetate-tolerance. In addition, the furfural- and phenol-tolerance of *E. coli* could also be enhanced by evolution approach. For example, an *E. coli* mutant EMFR9 obtained after 54 serial transfers under furfural concentration increasing from 0.5 to 1.3 g/L, could grow after a lag phase of < 48 h under the stress of lignocellulosic hydrolysate containing 0.42 g/L furfural (Miller et al. 2009b); and the microbiota resistance threshold of phenol can increase from 0.9 to 1.9 g/L after 3 successive disturbance episodes (Madigou et al. 2016).

Although it is well known that various inhibitors could exhibit synergistic toxicity, thereby increasing the inhibition effect of mixed inhibitors; our evolved strain *E. coli*-H was still highly tolerant to the combination of 1.4 g/L acetic acid, 1.6 g/L furfural, and 1.2 g/L phenol. The tolerance levels of the inhibitors are higher than or comparable to those of some previous strains specifically engineered for one single inhibitor like furfural and phenol. For example, a random combination of deletion of gene *yqhD* and increased expression of *fucO*, *ucpA*, or *pntAB* could help ethanologenic *E. coli* LY180 tolerate to 10–15 mM (equal to 0.96–1.44 g/L) furfural (Miller et al. 2009a, b; Wang et al. 2011, 2013); overexpression of whole phenol degradation pathway genes *pheA1*, *pheA2, catA*, *catB*, *catC*, *catD*, *pcaI*, *pcaJ,* and *pcaF* in *E. coli* BL221-AI resulted in a recombinant BL-*phe*/*cat* strain that could tolerate 10 mM (equal to 0.94 g/L) phenol (Wang et al. 2019). Thus, in view of the high inhibitor tolerance of the evolved strain *E. coli*-H, we proceeded to ferment the real cotton-derived bio-oil media by using this strain.

### Bioethanol production from undetoxified bio-oil by the evolved strains

Levoglucosan-to-ethanol fermentation performance of the evolved *E. coli*-H was conducted in bio-oil-based M9 minimal media, and the evolved *E. coli*-L was also used to ferment the bio-oil for comparison. The sugars and inhibitors in pyrolysis bio-oil can vary in a wide range with 1–305 g/L levoglucosan, 1–170 g/L acetic acid, 1–30 g/L furfural, and 1–38 g/L phenol; while levoglucosan between 6 and 150 g/L, acetic acid between 1 and 99 g/L, furfural between 1 and 11 g/L, and phenol between 1 and 7 g/L are most common (Islam et al. 2015; Milne et al. 1997). In our preliminary experiments, we detected 181.7 g/L levoglucosan, 11.4 g/L acetic acid, and 9.3 g/L furfural in the cotton-derived bio-oil without phenolic compounds (cotton is mainly composed of cellulose, while lignin is the main source of phenolic compounds). For the fermentation experiments, bio-oil was first diluted into the M9 salts solution by ~ 9-fold, resulting in a final levoglucosan concentration of ~ 20 g/L, which is the sugar concentration commonly used in laboratory-scale fermentation. The pH of the diluted bio-oil medium was measured as 3.1, and the acetic acid and furfural were diluted to ~ 1.3 and 1 g/L, respectively. In parallel, another weak acid, formic acid, which has similar toxicity to acetic acid, and another furan, 5-HMF, which is also functionally similar to furfural, were diluted to 0.3 and 1.7 g/L, respectively.

**Fig. 2:**
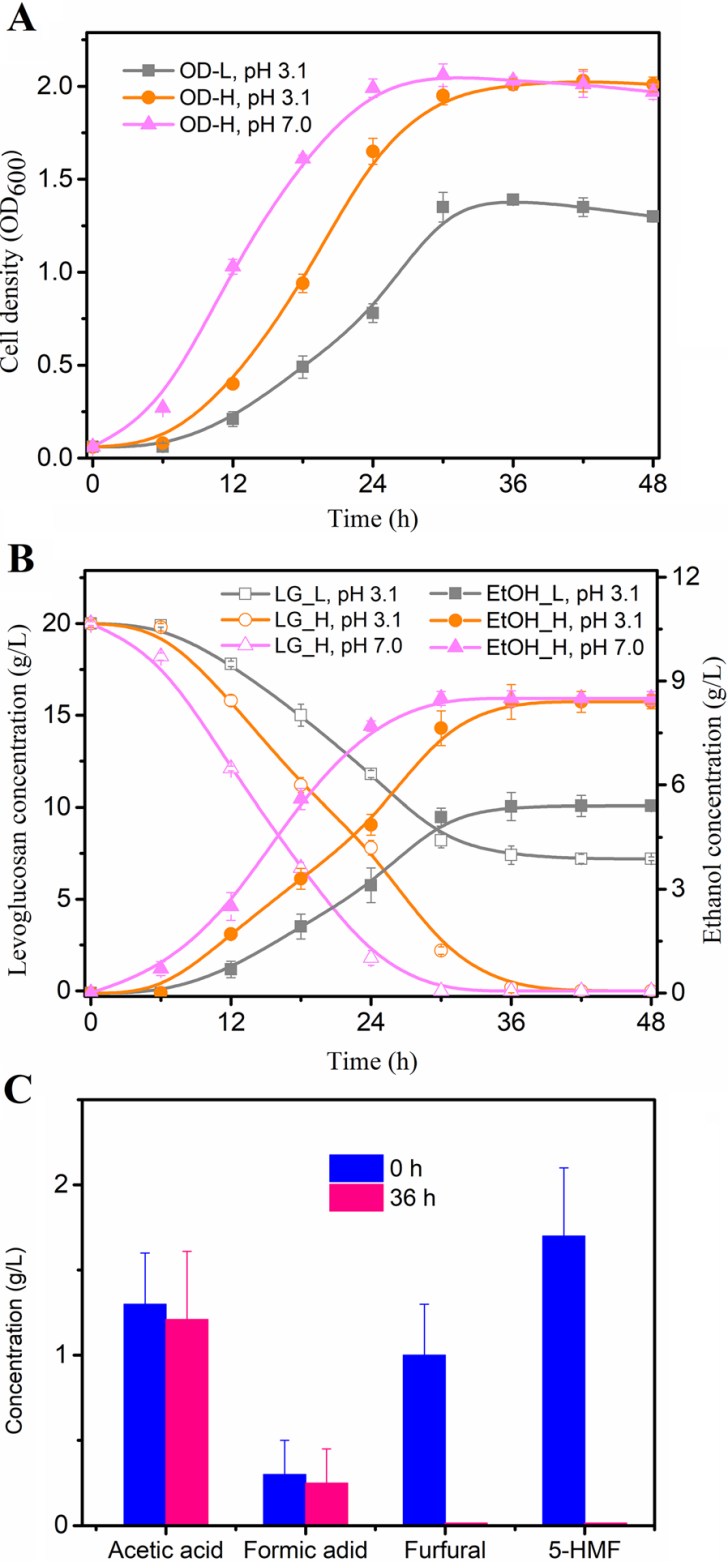
Cell growth, ethanol fermentation, and inhibitor conversion of the evolved *E. coli*-L and *E. coli*-H in bio-oil based minimal media. **A** Cell growth. OD-L and OD-H denote the OD_600_ values of *E. coli*-L and *E. coli*-H, respectively. **B** Levoglucosan consumption and ethanol production. LG-L and LG-H denote the levoglucosan residues after consumed by *E. coli*-L and *E. coli*-H, respectively, and EtOH-L and EtOH-H the respective ethanol concentrations produced by them.** C** Acetic acid, formic acid, furfural, and 5-HMF concentrations before (0 h) and after (36 h) fermentation. Conditions: 30 °C, 200 rpm, pH 3.1 or 7.0. Mean values were presented with error bars representing at least two standard deviations

The toxicity of weak acids, furans, and other toxic compounds present in the bio-oil can decrease with the increasing pH value, and overliming even can detoxify most of the inhibitors (Chi et al. 2013; Martinez et al. 2000). Therefore, we performed the fermentation tests under different pH conditions for comparisons. It should be noted that the original strain *E. coli* LGE2 could not grow and produce ethanol under any pH condition used here, because of the toxicity of bio-oil. Figure 2 exhibits the fermentation profiles of the evolved strains *E. coli*-L and -H. The growth rate and final cell density of *E. coli*-L in the bio-oil medium with a pH of 3.1 were lower than those of *E. coli*-H (Fig. 2A), consistent with the evolution result that *E. coli*-H was more tolerant to the inhibitors than *E. coli*-L (Fig. 1), although both strains reached the stationary growth phase at ~ 30-h time point (Fig. 2A). The sugar consumption of *E. coli*-L was negatively affected by low cell density, which subsequently resulted in inferior ethanol concentration to *E. coli*-H (Fig. 2B). In contrast with 7.2 g/L of residual levoglucosan left by *E. coli*-L at the end of the fermentation (initial pH of 3.1), *E. coli*-H consumed all the levoglucosan at ~ 36-h time point (*p* < 0.01) (Fig. 2B). As also shown in Fig. 2B, the faster levoglucosan metabolism in *E. coli*-H brought about a significantly higher ethanol generation of 8.4 g/L, 55.5% more than 5.4 g/L of ethanol was generated for *E. coli*-L with pH 3.1 (*p* < 0.05). However, although the ethanol yields of both strains were all ~ 0.42 g/g levoglucosan, the ethanol productivity of 0.28 g L^−1^ h^−1^ achieved by *E. coli*-H was much higher than that of 0.18 g L^−1^ h^−1^ achieved by *E. coli*-L. Furthermore, we also detected the changes in furfural, 5-HMF, acetic acid, and formic acid concentrations in the bio-oil media fermented by *E. coli*-H. Furfural and 5-HMF were all metabolized by *E. coli*-H, while acetic acid and formic acid had a slight change with respective 1.21 and 0.25 g/L left (Fig. 2C). This can be well explained by the facts that *E. coli* could 1) detoxify the furans (furfural and 5-HMF) to less toxic alcohols by its innate degradation pathway (Miller et al. 2009a; Wang et al. 2013), which might have been strengthened by the genetic mutations, and 2) tolerate the weak acids by other self-regulation ways like membrane efflux pump (Zhang et al. 2011) or amino acid-dependent acid-resistance system (Foster 2004; Xu et al. 2020) rather than the direct transformation mechanism.

As stated above, pH is strongly correlated to the toxicity of various inhibitors. Thus for more comparisons, we proceeded to use *E. coli*-H for subsequent ethanol fermentation after adjusting the initial pH of the bio-oil-based media from 3.1 to 7.0 by Ca(OH)_2_. As shown in Fig. 2B, under pH of 7.0, *E. coli*-H took less time to grow to the maximal cell mass, consume all the levoglucosan, and produce the maximal ethanol concentration than that taken under pH of 3.1 (24 vs 30 h), with the ethanol productivity of 0.35 g L^−1^ h^−1^ improved by 25% from ~ 0.28 g L^−1^ h^−1^. However, the values of final cell mass (OD_600_ 2.06 vs 2.03), ethanol concentration (8.5 vs 8.4 g/L), and ethanol yield (both 0.42 g/g levoglucosan) were similar. In terms of the obtained production indexes like cell mass, ethanol concentration, and ethanol yield other than ethanol productivity, our results showed that *E. coli*-H can perform as well in acidic pH as in neutral pH. Although at the expenses of productivity in low-pH media, our evolved strain *E. coli*-H could grow in and ferment the bio-oil without any costly detoxification or extraction procedures, thereby decreasing the overall costs.

Because the existence of other inhibitors could also inhibit the evolved strain to some extent, the cell growth rate, levoglucosan consumption rate, and ethanol production rate of the evolved strain were all slower than those obtained by the original strain *E. coli* LGE2 grown in pure levoglucosan-based minimal media (Chang et al. 2021). The final cell mass obtained by *E. coli*-H in bio-oil media was higher than that obtained by LGE2 in levoglucosan-based media (Chang et al. 2021), due to the different used UV spectrophotometer. However, ignoring the fact that inhibitors could result in a long lag phase during fermentation (Islam et al. 2015; Mills et al. 2009), the final ethanol yield and concentration obtained under the two conditions were similar, implying that our evolved strain could exhibit comparable fermentability in the toxic bio-oil to that of the original strain in the nontoxic levoglucosan-based media. More importantly, compared to the original strain that could not grow in the bio-oil media, the evolved strain *E. coli*-H could ferment all the levoglucosan substrate to ethanol. This is also significant progress compared to the previous researches (Chi et al. 2013; Rover et al. 2014), in which the bio-oil must firstly be chemically detoxified or separated by liquid extraction prior to fermentation, otherwise the cells would be killed by the inhibitors co-existing with levoglucosan.

Moreover, with the evolved tolerance, *E. coli*-H is also anticipated to be competitive in the bioconversion of another common lignocellulose-derived substrate (lignocellulosic hydrolysate), in which the common concentrations (without dilution) of total sugar (including xylose, glucose, arabinose, and mannose) is between 48.3 and 102 g/L, acetic acid is between 0.3 and 9 g/L, total furans is between 0.001 and 7.9 g/L, and total phenolic compounds is between 0.07 and 3 g/L (Duarte et al. 2005; Kurosawa et al. 2015; Martinez et al. 2000). For example, 0.38 g/L furfural, 0.21 g/L 5-HMF, 2.54 g/L acetic acid, 0.29 g/L vanillin, 0.13 g/L syringaldehyde, and 0.03 g/L 4-hydroxybenzaldehyde were present in the corn stover hydrolysate (Wang et al. 2018); and in another corn stover hydrolysate, the concentrations of furfural, 5-HMF and acetic acid were 0.3, 0.2, and 3.2 g/L, respectively (Jin et al. 2019); similarly, the concentrations of furfural, 5-HMF, and acetic acid were 0.6, 0.03, and 2.5 g/L in kenaf stems hydrolysate (Shah et al. 2020) and 7.9 g/L, 1.0 g/L, and 10.2 g/L in the wheat straw hydrolysate (Nielsen et al. 2015), respectively. The inhibitors with these concentrations could be tolerated by our evolved strain *E. coli*-H, after comparing the tolerance levels of *E. coli*-H as well as the inhibitor concentrations in bio-oil with the inhibitor concentrations present in the diluted lignocellulosic hydrolysate. Therefore, the robust strain *E. coli*-H developed by this study would be very useful in the cost-effective bioethanol production from the undetoxified lignocellulosic biomass-derived substrates, either bio-oil or hydrolysate.

### Mutations in *rssB*, *yqhA*, promoter of *yqhD-dgkA* operon, and *basR* contributed to the high inhibitor-tolerance of the evolved strain

To reveal how the evolved strains acquired inhibitor tolerance, comparative whole-genome re-sequencing was subsequently performed. The mean SNPs in an adapting *E. coli* genome was previously evaluated as 1.06 for every 100 generations (Dettman et al. 2012). In the current study, after a total of respective 302 and 374 generations, 3 and 4 SNPs were detected in the genome of *E. coli-*L and *E. coli-*H, respectively (Table 2). Notably, the three SNPs with genomic coordinates (referring to the reference genome of *E. coli* BL21 (DE3)) of 1,278,697, 3,016,868, and 3,022,732 were all found in *E. coli-*L and *E. coli-*H.

**Table 2:** Confirmed mutations discovered in two *E. coli* LGE2-evolved strains adapted to inhibitors-containing LB media

Strain	Gene	Product description	Location	Class	Nucleotide	Codon	Protein change
*E. coli*-L	*rssB*	Regulator of σ^S^ factor (RpoS)	Cytoplasm	Regulator	△T664	Frame shifts	L245 ⟶ stop
	*yqhA*	UPF0114 protein	Plasma membrane	Regulator analog	G41T	TGG ⟶ TTG	W14L
	IGR of *yqhC/yqhD*	5’ untranslated region of *yqhD*	Cytoplasm	Regulator (promoter)	T77C	AAT ⟶ AAC	− 10 box change
*E. coli*-H	*rssB*	Regulator of σ^S^ factor (RpoS)	Cytoplasm	Regulator	△T664	Frame shifts	L245 ⟶ stop
	*yqhA*	UPF0114 protein	Plasma membrane	Regulator analog	G41T	TGG ⟶ TTG	W14L
	IGR of *yqhC*/*yqhD*	5’ untranslated region of *yqhD*	Cytoplasm	Regulator (promoter)	T77C	AAT ⟶ AAC	− 10 box change
	*basR*	Transcription regulator	Cytoplasm	Regulator	T292C	TAT ⟶ CAT	Y98H

**Fig. 3:**
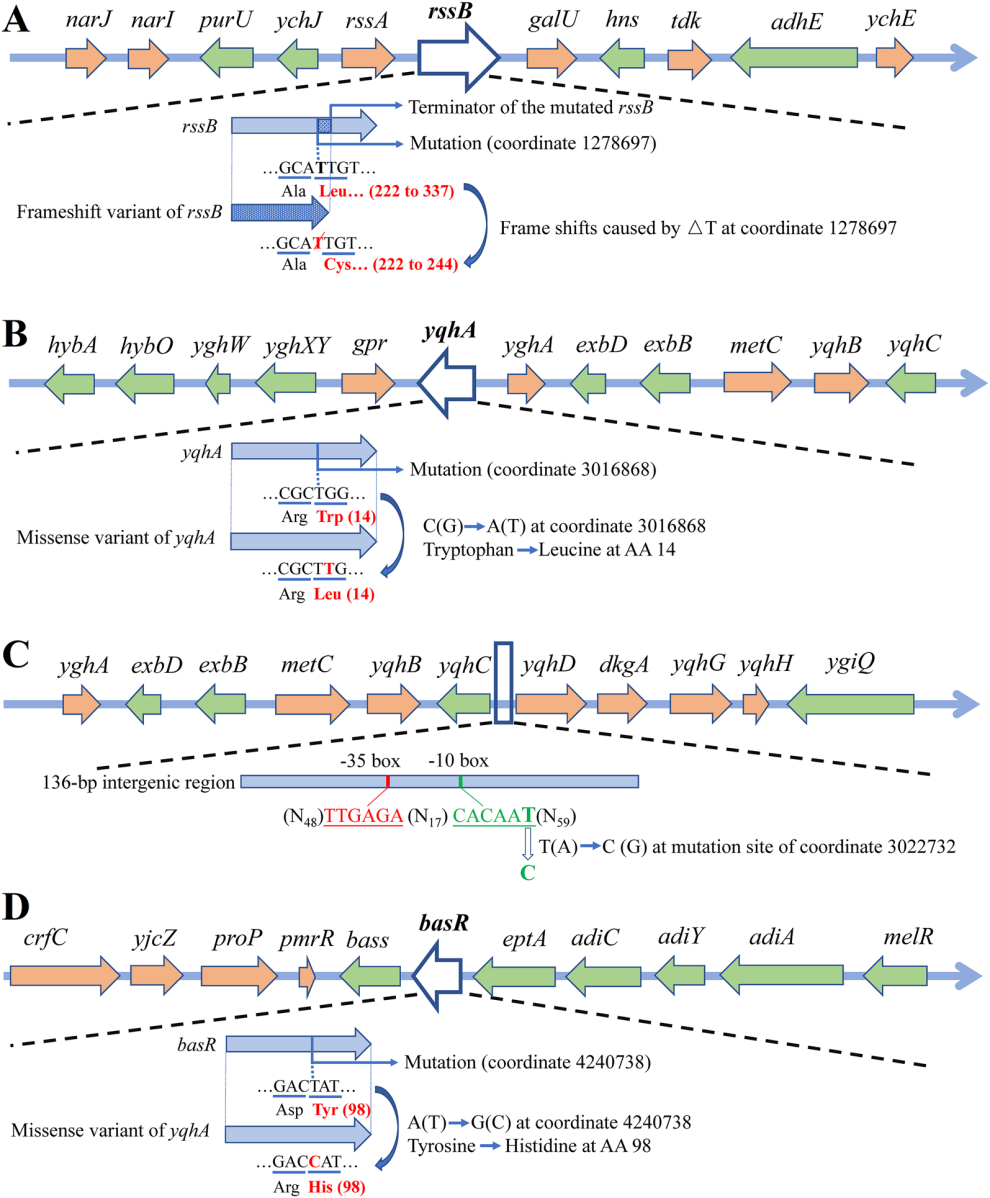
The mutations and mutation effects on gene expression/regulation. The surrounding genes annotated as upstream or downstream gene variant type by whole-genome sequencing are also shown here, with their directions on the genome. Mutation in *rssB* (**A**) caused the translational stop codon (TGA) to move forward, with a deletion of a total of 93 amino acids from the 245th position of RssB. Mutations in *yqhA* (**B**), intergenic region of *yqhC*-*yqhD* (**C**), and *basR* (**D**) resulted in missense variants. △ denotes deletion, and AA denotes amino acid

**Fig. 4:**
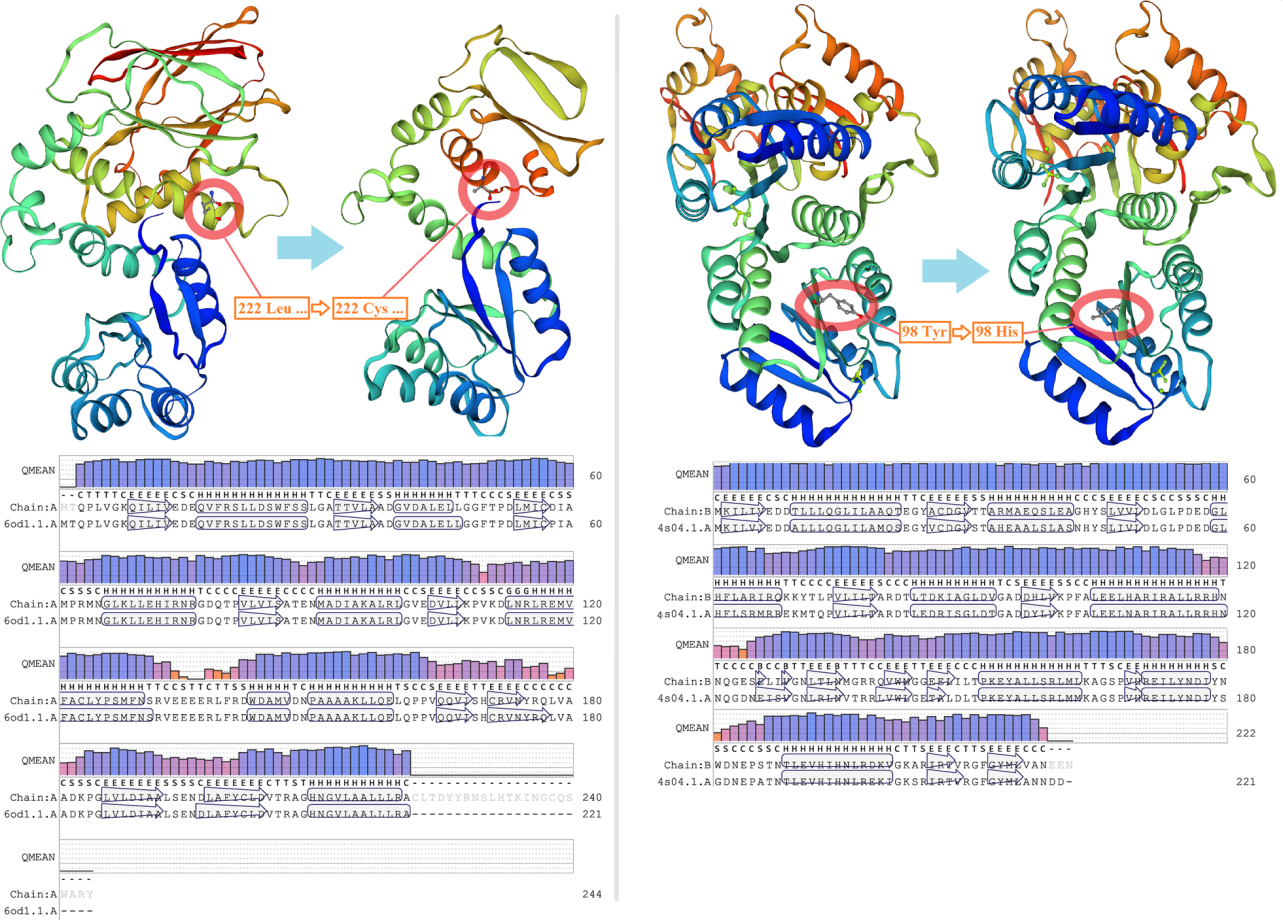
Three-dimensional structure and sequence alignment of the original and mutated proteins RssB (**A**) and BasR (**B**). The three-dimensional structure was modeled by SWISS-MODEL and modified by Chimera v_1.15 using a PDB file, showing a rainbow ribbon diagram, in which blue color denotes the starting point of the peptide and red stands for the endpoint. The mutation in RssB has occurred at the 222nd amino acid within the α-helix structure and in BasR is at the 98th amino acid within the β-sheet structure. The framed sequences shown in the sequence-alignment panels are involved in protein structures like α-helix, β-sheet, and β-turn

At position 1,278,697, the original base pair thymine in the genome was lost, resulting in a frameshift variant of *rssB* with the sequence change after 221st amino acid and a TGA terminator occurring after the 244th amino acid, thereby shortening the total amino acid length of RssB from 337 to 244 (Fig. 3A). For the structure analysis, 221 residues of the mutated RssB accounting for 244 aligned residues (PDB-entry: 6od1.1) were modeled by SWISS-MODEL at 2.00 Å with 100% confidence and 91% sequence coverage (Fig. 4A), which clearly showed the loss of peptides behind the 7th α-helix structure compared to the original RssB. Considering its function, RssB can negatively regulate RpoS via regulating the proteolysis of RpoS, which is the master regulator of *E. coli* that controls the expression of the general stress-response pathway. Apart from regulating RpoS, RssB also plays a multifunctional role in controlling transcriptome, adjusting polyadenylation, and modulating mRNA stability (Carabetta et al. 2009). It is known that the amino acid sequence of RssB from position 9 to 123 is a conserved region functioning as the response regulatory domain. Therefore, mutation of peptide sequences after the 222nd amino acid and deletion of a total of 93 amino acids from the 245th position of RssB would affect other unidentified domains of RssB and damage/modify the original structure and function of RssB. This is consistent with a previous report that loss of the *rssB* gene could result in a stabilization of RpoS and therefore enhance the levels of this stress-response sigma factor RpoS (Fontaine et al. 2008). Mutations in *rpoS* have been implicated in adaptation to many selective environments (Dettman et al. 2012); and also, mutations in *rssB*, which can alter the balance of cellular sigma factors and lead to elevated levels of RpoS, could lower the frequency of cell death and increase the cellular stress tolerance to a variety of external stresses including oxidative, osmotic, and solvent stress (Fontaine et al. 2008; Minty et al. 2011). Considering the RpoS-based general stress-response system can aid cells to respond to the endogenous stresses that are potentially fatal (Fontaine et al. 2008), it is undoubtedly that mutation obtained by adaptive evolution in the RpoS regulator RssB could confer benefits to the evolved strain for the acquirements of cellular inhibitor tolerance.

At position 3,016,868 within gene *yqhA*, the original base pair cytosine was altered to adenine, thus the 14th amino acid of YqhA was changed from tryptophan to leucine, causing a missense variant of YqhA (W14L) (Fig. 3B, Table 2). Tryptophan that prefers to be buried in protein hydrophobic cores can be substituted with other aromatic or hydrophobic amino acids. As a hydrophobic amino acid, leucine is also prone to be buried in protein hydrophobic core within α-helices (Betts and Russell 2003). The leucine side chain is non-reactive and thus rarely directly involved in protein functions like catalysis. However, it can play an important role in substrate recognition, especially in binding/recognition of hydrophobic ligands (Betts and Russell 2003) such as lipid, phenol, and furfural. This is consistent with the fact that YqhA located at the plasma membrane is a transmembrane protein, which is responsible for the signal transduction and the binding, recognition, and transport of chemicals. For YqhA, the amino acid sequence from position 9 to 125 is a domain feature that belongs to the uncharacterized protein family UPF0114, and next to the mutation site of position 14 is a helical transmembrane region from position 15 to 35. In addition to this known bioinformation, the structure and function of YqhA in *E. coli* remain unclear yet. Moreover, homology modeling of the mutated YqhA only showed 48% confidence with the highest scoring template-the Mrp antiporter complex (PDB-entry: 6z16c), therefore, the low confidence rendered us discard the constructed model. However, in *Bacillus subtilis*, *yqhA* has been identified as a paralog to *rsbR*, which encodes the positive regulator of sigma factor σ^B^ and functions in the environmental signaling branch of the general stress response (Akbar et al. 1997). This suggests a potential role of *E. coli* YqhA in stress modulation.

YqhC, as a transcriptional activator, can regulate the expression of genes *yqhD* and *dkgA*, which are NADPH-dependent oxidoreductase involved in the cellular tolerance to furfural and vanillin (Pattrick et al. 2019; Turner et al. 2011). In addition, alcohol dehydrogenase YqhD is also involved in the biodetoxification and bacterial survival in various aldehydes and other chemicals such as hydrogen peroxide, butanaldehyde, propanaldehyde, acrolein, malondialdehyde (Pérez et al. 2008), glutaraldehyde (Merchel Piovesan Pereira et al. 2021), and glyoxal (Lee et al. 2010). At position 3,022,732, which is located within the non-coding sequence (intergenic region) between the genes *yqhC* and *yqhD*, the original base pair thymine was altered to cytosine (Fig. 3C, Table 2). Functional elements like enhancer and promoter are usually located at the 5ʹ untranslated region of a certain gene. The intergenic sequence, 50 bp upstream of the open reading frame of *yqhD* and possessing a 24-bp palindrome consisting of two 10-bp repeating units, are predicted to be potential binding site sequences of *yqhC* to *yqhD-dkgA* operon (Frazao et al. 2018; Lee et al. 2010). The *yqhD* is preceded by the − 35 region TTGAGA and − 10 region CACAAT, to which the RNA polymerase and sigma factor σ^70^ bind to initiate transcription. The mutation site is located within the − 10 region of the predicted promoter of *yqhD* (Fig. 3C); therefore, this base pair substitution might alter the conformation and function of this binding site, thereby affecting the binding capacity of *yqhC* to the promoter of *yqhD-dkgA* operon. Taking our results together, this mutation was speculated to enhance the transcription strength of *yqhD* and *dkgA,* thereby improving the tolerance of the evolved strain to multiple lignocellulosic inhibitors.

*E. coli-*H was a strain evolved from *E. coli-*L. Compared to *E. coli-*L, one SNP in gene *basR* with a genomic coordinate of 4,240,738 was a new mutation found in *E. coli-*H; this mutation facilitated *E. coli-*L to be more tolerant to a higher concentration of inhibitors, implying the significance of this mutation to the cellular tolerance. At this position, the original base pair adenine was altered to guanine, resulting in the change of 98th amino acid of the gene *basR* from tyrosine to histidine (Y98H), thereby leading to a missense variant of *basR* (Fig. 3D, Table 2). Tyrosine is a partially hydrophobic amino acid prone to be involved in protein hydrophobic cores. A common role for tyrosine within intracellular proteins is phosphorylation, which is part of a signal transduction process (Betts and Russell 2003). However, histidine, as the most common amino acid in the protein active or binding sites, is a more ideal residue for protein functional center due to the flexibility that polar histidine is easy to move protons on and off from the side chain (Betts and Russell 2003). It is known that the two-component BasS/BasR signal transduction system controls a series of genes that are related to ion/metal-, low pH stress-, and anaerobic metabolism-responses (Hagiwara et al. 2004). Especially BasR, as an important part of the BasS/BasR system, has been reported to be involved in the cellular adaptations to complicated natural or artificial conditions (Hagiwara et al. 2004; Janssen et al. 2020; Rubin et al. 2015), and also in the regulation of the genes related to the modulation of membrane structure, membrane functions and stress responses in *E. coli* (Ogasawara et al. 2012). Moreover, regarding the structure of mutated BasR, homology modeling showed it had 100% confidence and 98% sequence coverage (217 residues aligned with 221 residues) at 3.20 Å with the highest scoring template-PmrA, a transcriptional regulatory protein from *Klebsiella pneumoniae* (PDB-entry: 4s04.1) (Fig. 4B). The amino acid sequence of BasR from position 2 to 116 is the response regulator domain and position 124 to 218 is the OmpR/PhoB-type DNA binding site. Compared to RssB that was mutated in the non-response regulator domain, the mutation position in BasR was within the response regulator receiver domain. Similarly, a single amino acid change (D28Y) from aspartic acid to tyrosine in position 28 within the response regulator receiver domain of BasR made the evolved *E. coli* LAR1 strain tolerate high C8 fatty acid stress (Chen et al. 2020) and the glycine in position 53 within this domain of BasR was also found altered in the colistin-resistant *E. coli* (Trent et al. 2001), implying that the phenotype acquirement by this site mutation in the response regulatory domain of BasR is owing to the alternation of stress response regulation.

It is noted that the four mutation sites (including three coding sequences *rssB*, *yqhA*, and *basR*, and one non-coding sequence between *yqhC* and *yqhD*) in the evolved strains are all implicated in the regulation function, implying that global regulation plays a key role in the cellular tolerance to lignocellulose-derived inhibitors. It has been previously reported that mutations in the transcriptase subunit RpoA of *E. coli* MEC136 (Rajaraman et al. 2016), in the transcriptional regulators FruR and McbR of *Corynebacterium glutamicum* (Wang et al. 2018), and in the putative promoter region of *mGDH* gene in *Gluconobacter oxydans* RM7 were found to be responsible for the cellular tolerance of lignocellulosic inhibitors (Jin et al. 2019).

Therefore, we strongly believe that these four mutated genes/elements were purposely chosen as biased targets of beneficial mutation by the strains themselves during the long-time adaptive evolution. Although we did not clearly present that to what extent each mutation contributed to the cellular inhibitor-tolerance acquired by adaptive evolution, we revealed that the four new mutations, especially the mutation in *basR*, could result in a more robust strain for bioethanol production from levoglucosan in the presence of various bio-inhibitors. It is also revealed that, for the first time, the gene *yqhA* and promoter region of *yqhD-dkgA* operon can be modified to enhance the cellular tolerance to multiple lignocellulosic inhibitors. The contribution of each mutation to the strains’ fitness improvement can be characterized in the future. Further genetic or protein engineering work on other microbial platforms by targeting these four regulatory elements revealed by this study could be expected to extend the production yield, titer, and efficiency of various bio-based products like biofuels and chemicals from the undetoxified lignocellulosic hydrolysate or pyrolysate with low cost.

## Conclusions

This work focused on both phenotypic and genotypic characterization of the evolved levoglucosan-utilizing ethanologenic *E. coli*, which was successfully developed by an adaptive evolution approach with high inhibitor tolerance and high bioethanol conversion efficiency from the undetoxified bio-oil. Under extremely low pH, the evolved strain *E. coli*-H could tolerate multiple inhibitors and ferment all the levoglucosan in the bio-oil to ethanol, with an ethanol production concentration and yield of respective 8.4 g/L and 0.42 g/g levoglucosan. Genome re-sequencing revealed the base-pair mutations within *rssB*, *yqhA*, promoter of *yqhD*-*dgkA* operon, and *basR* were responsible for the enhanced inhibitor tolerance and fermentation performance of the evolved strain. Further works that directly introduce the mutations in the original strain or reverting to the wild-type allele in the evolved strain, can be performed to fully understand the mechanisms involved in the phenotype alteration. However, the mutated genes revealed here can be considered as candidate genes for the genetic or proteinic modifications in other robust microbial catalysts in the future. The current study also provided a practical example for improving the microbial fermentability of cost-effective biofuel and other value-added chemicals in the presence of multiple inhibitors in industrial lignocellulose biorefinery.

## Data Availability

The datasets used and/or analyzed during the current study are available from the corresponding author on reasonable request.

## Abbreviations

MIC: Minimal inhibitory concentration
IC_50_: Half-inhibitory concentration
CIV: Concentration increase value
SNP: Single-nucleotide polymorphism
MNP: Multiple nucleotide polymorphisms
INS: Insertion
DEL: Deletion
MIXED: A combination of SNP, MNP, INS, and DEL
5-HMF: 5-Hydroxymethylfurfural
OD_600_: Optical density at wavelength (*λ*) 600 nm
HPLC: High-performance liquid chromatography
